# PET-imaging derived prognostic factors for prostate cancer patients with visceral metastases receiving [^177^Lu]Lu-PSMA radiopharmaceutical therapy (RPT)

**DOI:** 10.1007/s00259-025-07712-2

**Published:** 2026-01-13

**Authors:** Magdalena Sophie Späth, Helmut Dittmann, Richard Spallek, Eduardo Calderón, Jonas Mück, Andreas Brendlin, Steffen Rausch, Christian la Fougère, Nils F. Trautwein

**Affiliations:** 1 Department of Nuclear Medicine and Clinical Molecular Imaging, University Hospital Tuebingen, Otfried-Mueller-Str. 14, 72076 Tuebingen, Germany; 2 Department of Urology, University Hospital Tuebingen, Hoppe-Seyler-Str. 3, 72076 Tuebingen, Germany; 3 Department of Diagnostic and Interventional Radiology, University Hospital Tuebingen, Hoppe-Seyler-Str. 3, 72076 Tuebingen, Germany; 4 DFG Cluster of Excellence 2180 ‘Image-Guided and Functional Instructed Tumor Therapy’ (iFIT), University of Tuebingen, Roentgenweg 11, 72076 Tuebingen, Germany; 5 German Cancer Consortium (DKTK), German Cancer Research Center (DKFZ) Partner Site Tuebingen, Auf der Morgenstelle 15, 72076 Tuebingen, Germany

**Keywords:** [^177^Lu]PSMA, Radiopharmaceutical therapy, PET, Visceral metastases, Prostate cancer

## Abstract

**Introduction:**

Visceral metastases are a significant risk factor and may be the most critical determinant of outcome in patients with metastatic castration-resistant prostate cancer (mCRPC) undergoing [¹⁷⁷Lu]Lu-PSMA RPT. In this monocentric real-world study, we evaluated baseline parameters derived from PET scans prior to RPT in order to identify potential prognostic factors in mCRPC patients with visceral metastases.

**Materials and methods:**

This retrospective study included 320 men with mCRPC treated with in total 1077 cycles of [^177^Lu]Lu-PSMA RPT (1–12 cycles/patient). Baseline characteristics, including age, PSA level, Gleason score, metastatic sites, and prior treatments, were evaluated as potential prognostic factors in the overall cohort. 65 patients were diagnosed with visceral metastases. In this subgroup, we analyzed the PET-derived parameters whole body PSMA-positive tumor volume (PSMA-TV), SUVmax, SUVmean, miPSMA expression score and the metastatic pattern of visceral metastases.

**Results:**

The estimated median overall survival (OS) for the entire cohort was 15 months. Presence of visceral metastases, Gleason score, and PSA level were significant negative prognostic baseline factors for the entire cohort in the multivariate Cox regression score. The estimated median OS for patients with visceral metastases was 10 months, which was significantly lower than the 16 months observed for patients without visceral metastases. PSMA-TV, miPSMA expression score, and the metastatic pattern were identified as independent prognostic factors in multivariate Cox regression analysis. Patients exhibiting two risk factors or more demonstrated a significantly poorer OS of 4 months compared to patients with a maximum of one risk factor, who showed an OS of 15 months.

**Conclusion:**

This study confirmed that visceral metastases represent a major risk factor in patients with mCRPC undergoing [^177^Lu]Lu-PSMA RPT. Moreover, PSMA-PET-derived parameters assessed prior to RPT enabled the distinction between high-risk and low-risk subgroups among patients with visceral metastases. These prognostic factors may therefore contribute to improved therapy stratification in mCRPC patients with visceral metastases undergoing [^177^Lu]Lu-PSMA RPT.

**Supplementary Information:**

The online version contains supplementary material available at 10.1007/s00259-025-07712-2.

## Introduction

Significant advances in recent years have expanded the therapeutic landscape for metastatic castration-resistant prostate cancer (mCRPC) [1, 2]. A particularly promising development has been the introduction of ^177^Lutetium (^177^Lu)-labeled compounds for radiopharmaceutical therapy (RPT) targeting the prostate specific membrane antigen (PSMA). Since the approval of (^177^Lu)-Lutetiumvipivotide tetraxetan ([^177^Lu]Lu-PSMA-617) by both the Food and Drug Administration (FDA) and the European Medicines Agency (EMA), RPT with [^177^Lu]Lu-PSMA has become an established component of clinical management in patients with mCRPC who had progressed despite treatment with androgen receptor signaling inhibitors (ARSI) and taxane-based chemotherapy [3, 4]. For this patient population, the pivotal VISION trial demonstrated that adding [^177^Lu]Lu-PSMA-617 to the standard of care (SoC) significantly improved both the progression-free survival (PFS) and overall survival (OS) compared with SoC alone [5]. Owing to the heterogeneity in treatment response among patients with mCRPC receiving RPT, as well as the broad range of alternative approved therapies, treatment selection should be individualized to optimize patient outcomes. Consequently, the identification of risk factors in patients undergoing [^177^Lu]Lu-PSMA-617 RPT represents an important area of ongoing research [6–8]. To identify sufficient PSMA-targeting in candidates for RPT, positron emission tomography (PET) imaging is routinely performed prior to therapy. Currently, different PET-ligands radiolabeled with Ga-68, F-18 or Zr-89 [9–12] are used for the non-invasive assessment of PSMA-positive tumor and metastases. For two widely used radiotracers [^68^Ga]Ga-PSMA-11 and [^18^F]PSMA1007 different comparative studies revealed consistent results for the lesion detection [13, 14].

SUV-based metrics as well as the PSMA-positive tumor volume (PSMA-TV) were shown to be non-invasive prognostic biomarkers in patients undergoing [^177^Lu]Lu-PSMA RPT [15–18]. Despite PSMA imaging, FDG PET performed prior to [^177^Lu]Lu-PSMA RPT was proven to improve therapeutic stratification in the TheraP study: a higher PSMA SUVmean (≥ 10) was identified as a positive predictive biomarker for OS; however a high metabolic tumor volume on FDG PET was associated with a worse OS [19, 20].

Further previous studies have demonstrated that visceral metastases represent a major and possibly the most important risk factor in patients undergoing [^177^Lu]Lu-PSMA RPT [21–25]. Consequently, appropriate therapeutic stratification for this patient subpopulation is of particular importance. Within this cohort, it is essential to identify which patients are likely to benefit from [^177^Lu]Lu-PSMA RPT, thereby sparing non-responders from unnecessary hospitalization and treatment-related toxicity, while guiding them toward alternative therapeutic options.

The present study therefore aimed to evaluate baseline PET-derived parameters as potential prognostic markers in mCRPC patients with visceral metastases prior to [^177^Lu]Lu-PSMA RPT.

## Materials & Methods

### Study population and clinical patient characteristics

The local database was retrospectively screened for mCRPC patients treated with [^177^Lu]Lu-PSMA between January 2018 and December 2024. In this study, 320 consecutive men with PET diagnosed PSMA-positive mCRPC at the University Hospital Tuebingen were included. Treatment decisions were made by an interdisciplinary tumor board, either within the framework of compassionate use or as part of clinical routine care. Patients gave their written consent after being informed about side effects and risks of [^177^Lu]Lu-PSMA RPT. The local ethics committee approved this study (approval 488/2025BO2), which waived the requirement for additional patient consent. The study was performed in accordance with the Declaration of Helsinki. Demographic variables were collected for all patients, including Gleason score, age, baseline prostate-specific antigen (PSA) values, TNM status before [^177^Lu]Lu-PSMA RPT. The best PSA change was evaluated according to the Prostate Cancer Working Group 3 [26].

### PET imaging and radiopharmaceuticals

 [^18^F]PSMA-1007 and [^68^Ga]Ga-PSMA-11 were synthesized, as described previously [12, 27]. Whole body PET imaging was performed on three different PET scanners: on a conventional PET/CT (Siemens Biograph mCT; PET data acquisition with continuous bed motion of 0.7 mm/s), on a PET/MRI scanner (Siemens mMR; PET data acquisition time of 4 min per bed position) and on a long–axial-field-of-view scanner (Siemens Biograph Quadra; PET data acquisition time of 5 min at a single bed position). PET with [^18^F]PSMA-1007 was acquired after a mean uptake time of 85 ± 14 min and a mean uptake time of 60 ± 4 min for [^68^Ga]Ga-PSMA-11. PET data reconstruction was performed, as described previously [12, 28–30].

All PSMA PET scans were acquired prior to [^177^Lu]Lu-PSMA RPT. PSMA I&T acetate (GMP) was purchased from ABX and [^177^Lu]LuCl3 in n.c.a. quality was provided as EndolucinBeta^®^ by ITM Pharma Solutions GmbH (Garching, Germany) for the preparation of [^177^Lu]Lu-PSMA-I&T [27]. [^177^Lu]Lu-PSMA-617 was provided by Novartis Radiopharmaceuticals. All patients received with at least one and up to twelve cycles of [^177^Lu]Lu-PSMA RPT in 6–10 week intervals. [^177^Lu]Lu-PSMA RPT was administered through a slow intravenous infusion in a 30-min time period followed by a saline solution.

### PET imaging analysis

Image analysis was performed in consensus by two readers for all scans using the dedicated software Affinity Hybrid Viewer (Version 3.0.5, Hermes Medical Solution, Sweden). The PSMA-TV was calculated by threshold-based semi-automatic volumetric segmentation applying the threshold described by Gafita et al. for ^68^Ga-labelled PSMA tracers [31]. We adapted the segmentation approach, as patients with lung and liver metastases could not be reliably segmented, particularly when [^18^F]PSMA-1007 was used. For liver metastases, segmentation was performed using a threshold of 1.5 × SUVmean + 2 × SD (standard deviation) of healthy liver tissue, as previously described, and for lung metastases 1.5 × SUVmean + 2 × SD of the blood pool was applied.

$$\:SUVthr=\frac{4.30}{SUVmean}\times\:\:(SUVmean+SD)$$

For all patients, the visceral metastatic pattern was distinguished as oligometastatic or disseminated. An oligometastatic pattern was defined as the presence of less than six metastatic lesions consistent with the criteria applied by Deek et al. [32, 33]. Visceral metastases were scored according to their PSMA expression using the miPSMA expression score [34].

### Visceral metastases risk score

Patients received one point for each of the following risk factors: a disseminated visceral metastatic pattern, at least a visceral metastasis with a miPSMA expression score < 3, and a PSMA-TV higher than the median for this cohort (746.3 ml).

### Statistical analysis

Baseline data were summarized as means ± SD. Survival hazards were estimated using univariate or multivariate Cox regression analyses. Due to their skewed distribution, the quantitative variables PSA and PSMA-TV were converted into log 10, as previously described by others [35–37]. Survival analysis was performed using Kaplan-Meier curves and Log-Rank tests. OS was defined as the time from the first treatment cycle to death in months*.* Statistical analyses and figures were performed using either GraphPad Prism (Version 9.4.1, GraphPad Software, San Diego, CA, USA) or SPSS (Version 28.0.0.0).

## Results

### Patient characteristics

**Fig. 1:**
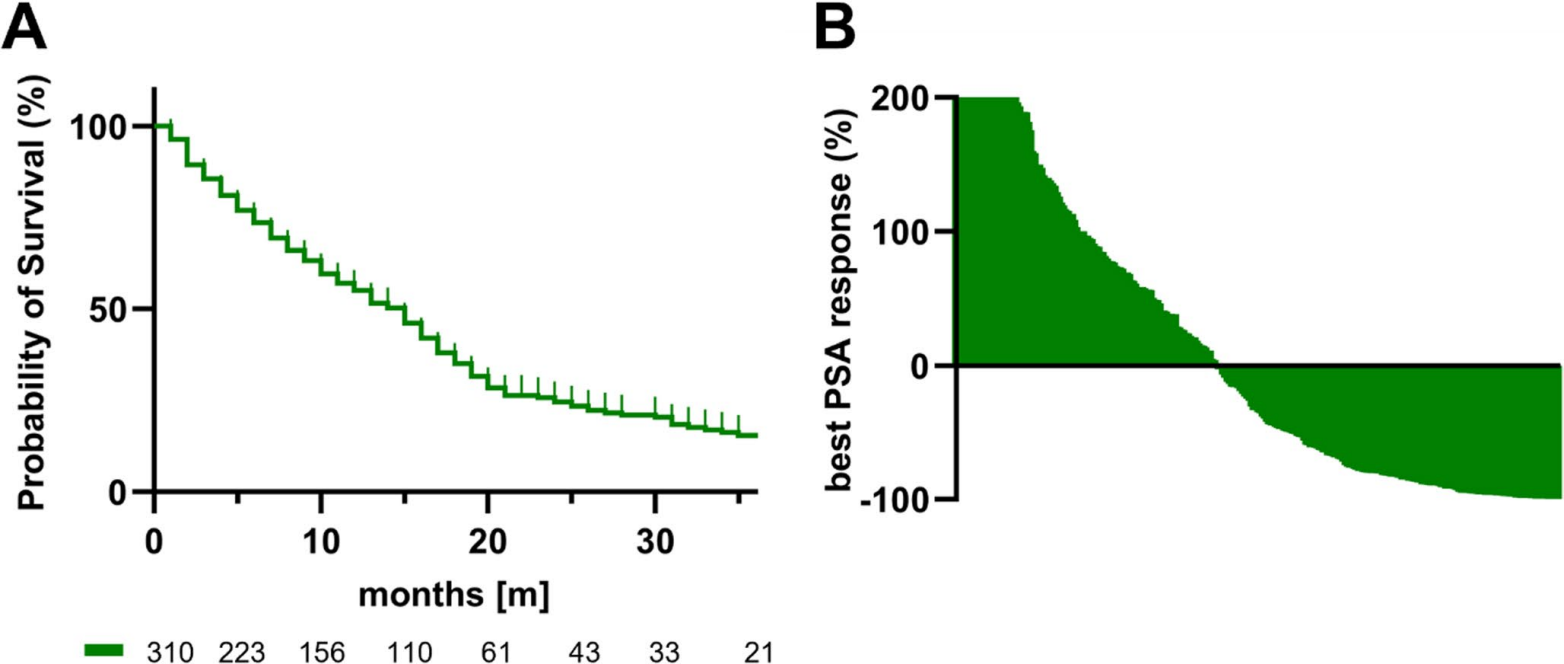
Kaplan-Meier curve of the OS in the entire mCRPC cohort, with an estimated median OS of 15 months (**A**). Waterfall plot depicting the best PSA change (%) from baseline over the total follow-up period; PSA increases > 200% were truncated for clarity (**B**)

320 male patients with mCRPC undergoing a total of 1077 cycles of [^177^Lu]Lu-PSMA RPT (mean: 3.37, 1–12) were included in this study. 65, approximately 20%, of patients presented with visceral metastases. The baseline characteristics of mCRPC patients receiving [^177^Lu]Lu-PSMA RPT are shown in Table 1. The estimated median OS of this entire cohort was 15 months (Fig. 1A). Subsequent to RPT, 49.1% of the patients showed any decline in PSA levels, while a PSA decline of ≥ 50% was observed in 38.4% and a PSA decline of ≥ 90% in 18.1%. The best change of PSA is displayed in Fig. 1B.

**Table 1:** Patient characteristics

Characteristics	*N*	Entire cohort (*n* = 320)	Visceral metastases (*n* = 65, 20%)	Non-visceral metastases (*n* = 255, 80%)
Age (y)	320	38–92	73–85	38–92
PSA before treatment (µg/L)	318	0.15–12,530	0.15–12,530	0.33–3125
Gleason score	288			
6		7 (2%)	2 (3%)	5 (2%)
7		57 (20%)	16 (25%)	41 (17%)
8		66 (23%)	14 (22%)	52 (20%)
9		131 (45%)	26 (40%)	105 (41%)
10		27 (9%)	2 (3%)	25 (10%)
Metastatic site at PSMA treatment	320			
N1 and M0		*3 (1%)*	*0 (0%)*	*3 (1%)*
M1a		14 (4%)	0 (0%)	14 (5%)
M1b		238 *(74%)*	0 (0%)	238 (93%)
M1c		65 (20%)	65 (100%)	0 (0%)
Bone involvement*		*296*	*59*	*237*
Unifocal		*7 (2%)*	*2 (3%)*	*5 (2%)*
Oligometastatic		*14 (5%)*	*7 (12%)*	*7 (3%)*
Disseminated		*202 (68%)*	*29 (49%)*	*173 (73%)*
Diffuse marrow involvement		*73 (25%)*	*21 (36%)*	*52 (22%)*
Pre-treatment	320			
Surgery		177 (55%)	38 (58%)	139 (55%)
Radiatio		170 (53%)	37 (57%)	133 (52%)
ADT		319** (100%)	65 (100%)	254** (100%)
ARSI		297 (93%)	57 (88%)	240 (94%)
Docetaxel		242 (76%)	46 (71%)	196 (77%)
Cabazitaxel		97 (30%)	27 (42%)	70 (27%)
Radium		28 (9%)	5 (8%)	23 (9%)
PARP		15 (5%)	1 (2%)	14 (5%)
Other chemotherapies		6 (2%)	2 (3%)	4 (2%)
Treatment line in mCRPC	320			
1. line		5 (2%)	0 (0%)	5 (2%)
2. line		41 (13%)	12 (18%)	29 (11%)
≥ 3. line		274 (86%)	53 (82%)	221 (87%)
PRLT	320			
Numbers of cycles		1–12	1–8	1–12
Dose		5495–8588	5945–8342	5495–8588
PSA response (≥ 50%)		123 (38%)	24 (37%)	99 (39%)

*The skeletal tumor burden was assessed according to PROMISE V2 [34]

**1 patient declined ADT

### Identification of patients prone to shorter survival through baseline characteristics

In the entire cohort, PSA and the presence of visceral metastasis, were identified as significant predictors of OS by univariate Cox regression analysis. Gleason score and previous therapies had p values < 0.1 and were included in the multivariate analysis. Age was excluded because it revealed a p value > 0.1 (Table 2).

**Table 2:** Univariate and multivariate Cox-Regression analysis of potential risk factors in the entire mCRPC cohort

Parameter	Univariate				Multivariate			
	*p*	HR	95% CI		*p*	HR	95% CI	
			Lower	Upper			Lower	Upper
Age (Y)	0.14	0.99	0.97	1.00				
PSA (µg/mL)	< 0.01	1.71	1.44	2.03	< 0.01	1.69	1.40	2.04
Gleason	0.09	1.13	0.98	1.29	0.02	1.19	1.03	1.37
Metastases (visceral/ non-visceral)	0.02	1.45	1.06	1.99	0.01	1.61	1.15	2.21
Pre-treatment	0.06	1.19	0.99	1.43	0.45	1.08	0.88	1.32

**Fig. 2:**
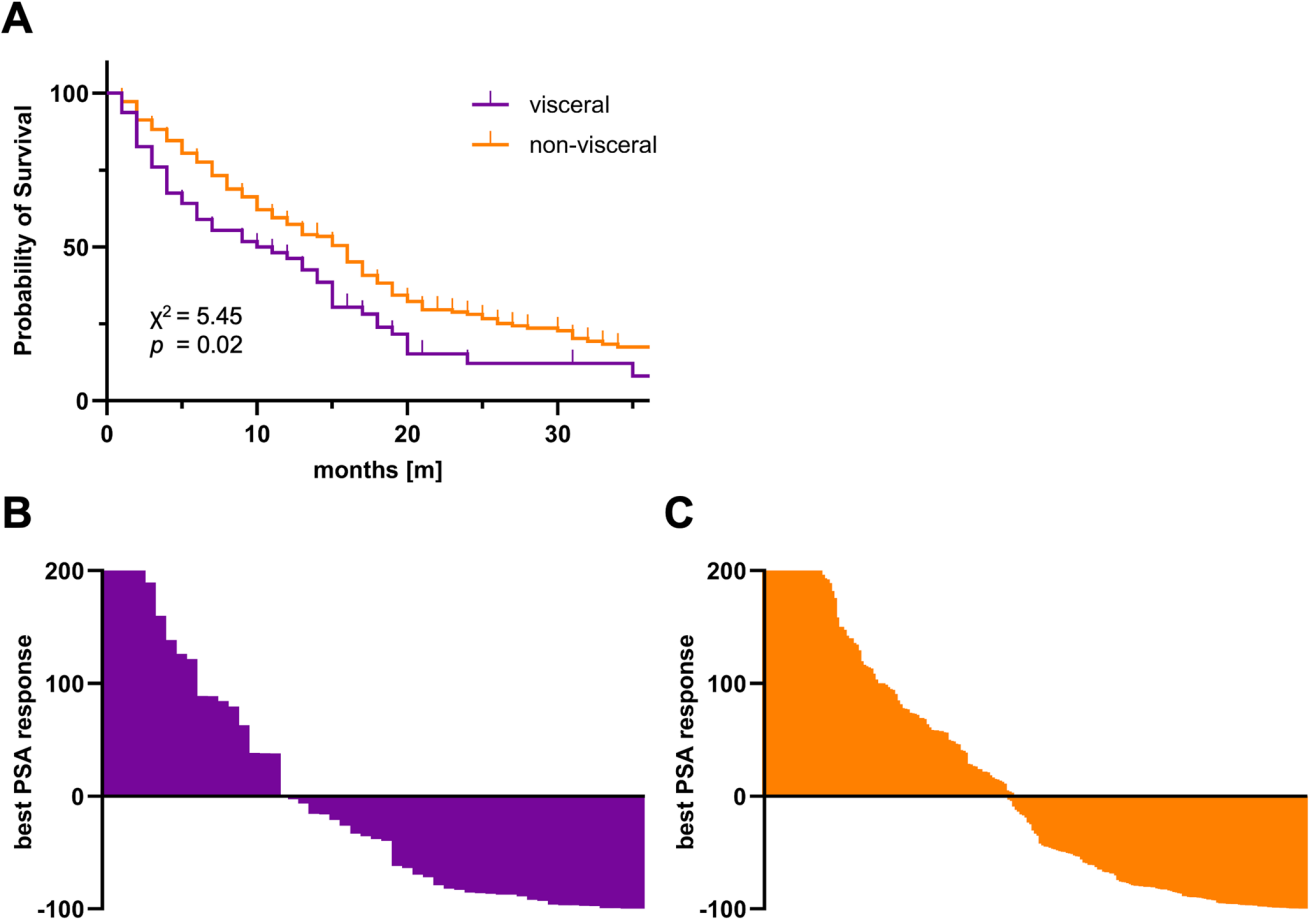
Kaplan-Meier curves of OS in mCRPC patients with and without visceral metastases, showing a significantly shorter median OS in patients with visceral metastases (10 vs. 16 months) (**A**). Waterfall plots of best PSA change (%) from baseline over the total follow-up period are shown for patients with visceral metastases (**B**) and without visceral metastases (**C**). PSA increases > 200% were truncated for clarity

Multivariate Cox regression analysis revealed significant hazard ratios (HR) for PSA of 1.69 (95% CI, 1.4–2.04), presence of visceral metastases of 1.61 (95% CI, 1.15–2.25) and Gleason score of 1.19 (95% CI, 1.03–1.37), while number of previous treatment lines were not significant (Table 2). Patients with visceral metastases had a significantly shorter OS rate of 10 months compared to 16 months in patients without visceral metastases (*p* = 0.02; Fig. 2A). Among the patients with visceral metastases, 53.9% exhibited any PSA decline, while a decline ≥ 50% was observed in 36.9% and a PSA decline ≥ 90% in 16.9%. The best PSA response is shown in Fig. 2B for patients with visceral metastases and in Fig. 2C for those without.

### PET-derived baseline parameters prognostic of survival after [¹⁷⁷Lu]Lu-PSMA RPT in visceral metastatic disease

PSMA-TV, metastatic pattern and miPSMA score of visceral metastases showed significant HR in the univariate Cox regression analysis. SUVmean and SUVmax were excluded for the multivariate analysis because of p values > 0.1 (Table 3).

PSMA-TV (HR = 1.97; 95% CI, 1.18–3.27, *p* = 0.01) and metastatic pattern (HR = 2.06; 95% CI, 1.15–3.71, *p* = 0.02) were identified as significant negative predictors in the multivariate Cox regression analysis. Patients with an oligometastatic pattern had significantly longer OS compared to those with a disseminated metastatic pattern (Supplementary Fig. 1A). Similarly, patients with a PSMA-TV below the median experienced significantly longer OS than those with a PSMA-TV above the median (Supplementary Fig. 1B). In contrast, a higher visceral metastases miPSMA score was a significant positive predictor (HR = 0.47; 95% CI, 0.28–0.80), with patients scoring 3 tending towards longer OS than those with a score of 2 or 1 (Supplementary Fig. 1C).

**Fig. 3:**
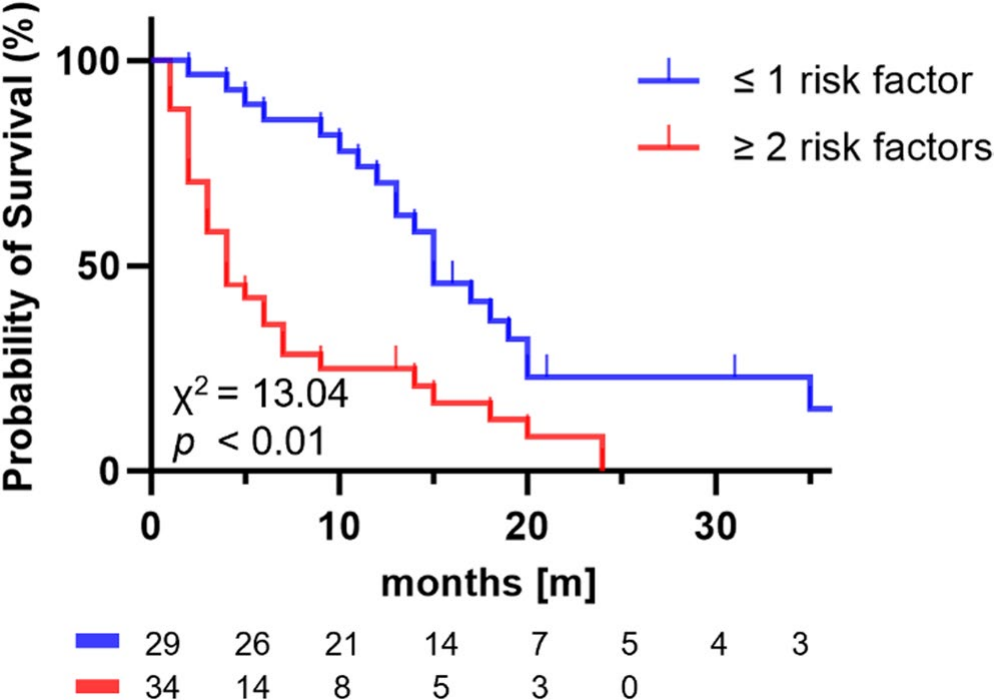
Kaplan-Meier curves of OS in patients with visceral metastases stratified by number of risk factors. Patients with ≥ 2 risk factors had a significantly shorter median OS (4 months) compared to those with 1 or no risk factor (15 months)

Patients with 1 or no risk factor had a significantly longer median OS of 15 months compared to those with ≥ 2 risk factors (4 months; *p* = 0.01; Fig. 3).

There was a trend towards shorter OS in patients with liver metastases; however, there was no significant difference according to the site of visceral metastases (Supplementary Fig. 2).

## Discussion

This study assessed the efficacy of [^177^Lu]Lu-PSMA RPT and prognostic factors for OS in patients with mCRPC and visceral metastases.

The median OS of 15 months in our complete cohort was comparable to that reported in the Vision trial [5]. Several studies have already emphasized the prognostic impact of visceral metastases [21–25], and our findings further confirm that visceral metastases represent a major risk factor in mCRPC patients. Therefore, appropriate treatment selection for this subpopulation is crucial, and a more personalized therapeutic approach is desirable. Thus, we evaluated PSMA PET-derived parameters as potential decision-making tools and demonstrated that the visceral metastatic pattern, the miPSMA expression score of visceral metastases, and PSMA-TV significantly affected OS in multivariate Cox analysis. Combining these risk factors enabled the identification of a substantial subgroup of patients with visceral metastases who may truly benefit from [^177^Lu]Lu-PSMA RPT. Notably, patients with up to one risk factor achieved a median OS of 15 months, comparable to that of patients without visceral metastases (16 months).

Conversely, this study demonstrated that patients with two or three PET-derived risk factors had a median OS of only 4 months. For this high-risk subgroup with visceral metastases, the indication of [^177^Lu]Lu-PSMA RPT should therefore be carefully reconsidered. Treatment decisions should always be based on interdisciplinary consensus, and alternative therapeutic strategies including more potent radionuclide emitters such as ^225^Actinium might be taken into consideration [38, 39]. Nevertheless, even within this high-risk group, a small number of patients achieved an OS of > 15 months indicating that [^177^Lu]Lu-PSMA RPT may still represent a viable treatment option. In such cases, the indication should be made on an even more individualized level, including clinical parameters such as ECOG performance score, age, body mass index, blood parameters and comorbidities [40–42]. In selected cases, avoiding further hospitalizations and instead prioritizing time with family and friends may be more meaningful for the patients. Thus, SoC should also be considered a valid therapeutic option in this setting.

Our findings are in line with studies on [^177^Lu]Lu-PSMA RPT in patients without visceral metastases. Seifert et al. demonstrated that the PSMA-TV is a prognostic risk factor in patients undergoing [^177^Lu]Lu-PSMA RPT [17]. Furthermore, several studies could demonstrate that the PSMA expression is an important predictor for therapy response [16, 43, 44]. Patients with mCRPC and visceral metastases represent a particularly vulnerable subpopulation, underscoring the need for specific decision-making tools. In this study we demonstrated that three easily assessable PET-derived risk factors, readily available from routine baseline PET, can improve patient stratification.

These findings may help to identify the most suitable candidates for [^177^Lu]Lu-PSMA RPT, ensuring that patients with mCRPC and visceral metastases receive the most appropriate treatment. This is of particular importance given the promising results of recent phase II and III trials, which suggest that the indications for [^177^Lu]Lu-PSMA RPT may be expanded to earlier treatment lines [45, 46]. However, such expansion will inevitably increase demands on therapeutic capacity.

This study has some limitations. First, it is limited by its retrospective and monocentric design. Furthermore, the same analytical approach for the assessment of PET based PSMA-TV was applied to both tracers, which however come with different tracer kinetics due to a liver dominant excretion of [^18^F]PSMA-1007 and a renal excretion of [^68^Ga]Ga-PSMA-11.

## Conclusion

Visceral metastases remain a major risk factor in patients with mCRPC undergoing [^177^Lu]Lu-PSMA RPT. In this study, we identified three PET-derived risk factors, the PSMA-TV, the pattern of visceral metastases and a visceral metastases miPSMA expression score < 3. Patients with visceral metastases and one or no risk factor showed a median OS comparable to that of patients without visceral metastases. As these parameters can be readily assessed from routine PET imaging, they may be rapidly implemented into clinical decision-making.

**Table 3:** Univariate and multivariate Cox-Regression analysis of potential risk factors in mCRPC patients with visceral metastasis

Parameter	Univariate				Multivariate			
	*p*	HR	95% CI		*p*	HR	95% CI	
			Lower	Upper			Lower	Upper
PSMA-TV	0.01	1.96	1.18	3.24	0.01	1.97	1.18	3.27
miPSMA	0.05	0.64	0.41	1.01	0.01	0.47	0.28	0.8
SUVmean	0.23	0.96	0.91	1.02				
SUVmax	0.97	1.0	0.99	1.01				
Metastatic pattern (oligo/ disseminated)	0.01	2.19	1.24	3.86	0.02	2.06	1.15	3.71

## Supplementary Information

Below is the link to the electronic supplementary material.

Supplementary Material 1 (DOCX 150 KB)
