# Supplementary material for: PET-imaging derived prognostic factors for prostate cancer patients with visceral metastases receiving [177Lu]Lu-PSMA radiopharmaceutical therapy (RPT)
Source: Eur J Nucl Med Mol Imaging. 2026 Jan 13;53(6):3590–7. doi: 10.1007/s00259-025-07712-2 (PMC13121192; doi:10.1007/s00259-025-07712-2)
Supplement: Supplementary file 1 — Supplementary Material 1 (DOCX 150 KB) [file 259_2025_7712_MOESM1_ESM.docx]

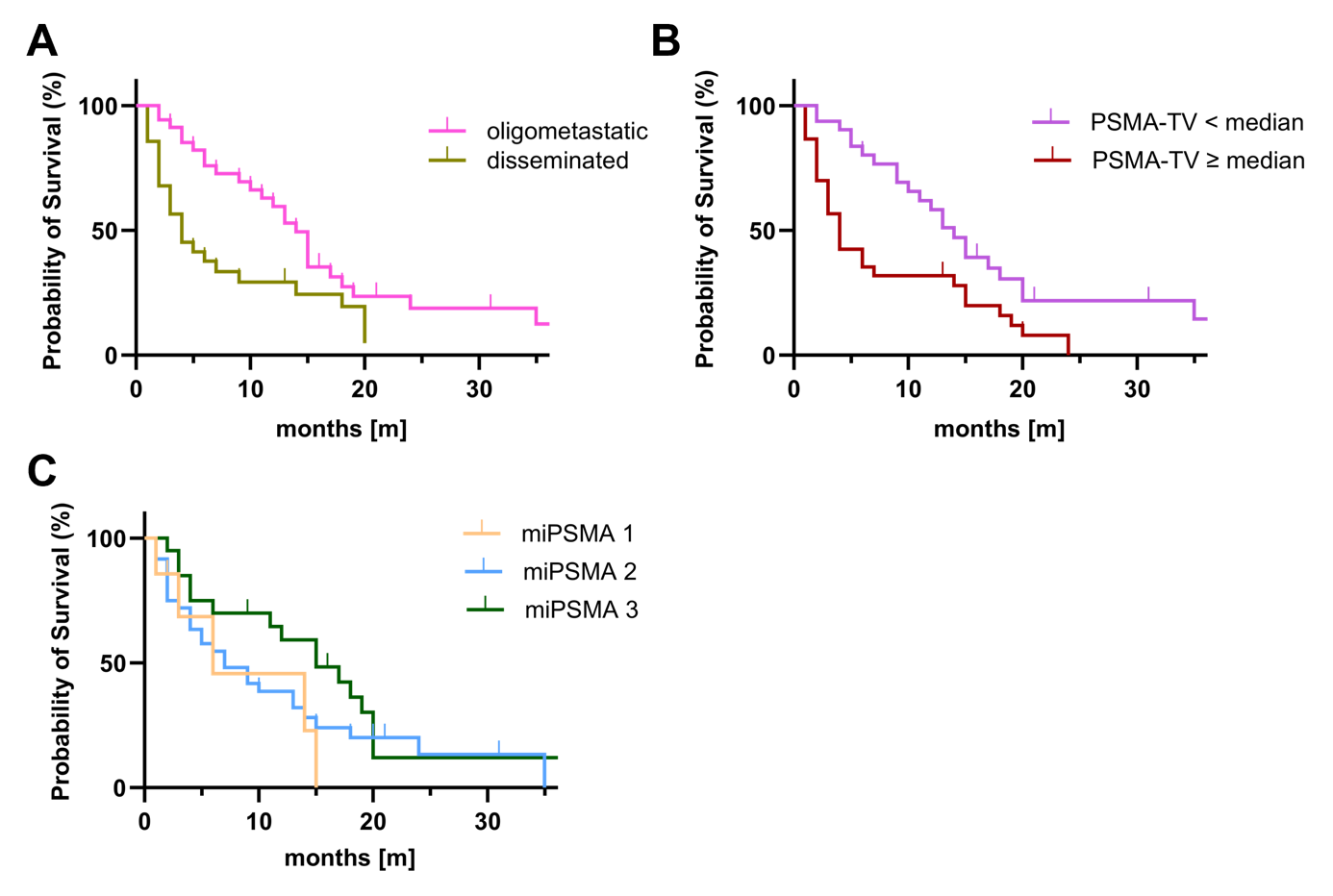


**Supplementary Figure 1:** Kaplan-Meier curves of OS in patients with visceral metastases categorised by risk factors. **(A)** Stratified by metastatic pattern: oligometastatic disease showed a median OS of 14 months, while disseminated disease showed a median OS of 4 months. **(B)** Stratified by PSMA-TV: patients below the median PSMA-TV had a median OS of 14 months, whereas those above the median had a median OS of 4 months. **(C)** Stratified by miPSMA expression score.


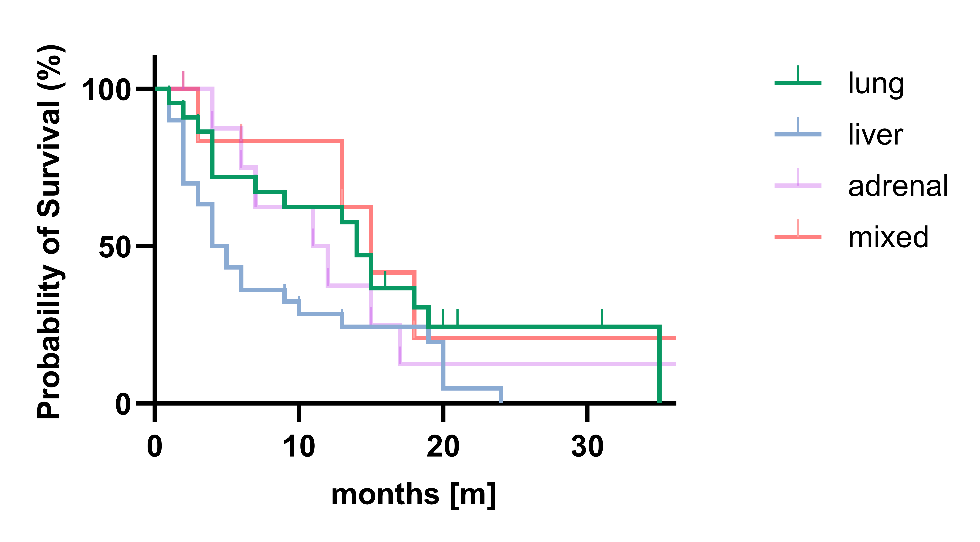


**Supplementary Figure 2:** Kaplan-Meier curve of OS in patients with visceral metastases stratified by the site of visceral metastases (non-significant; p = 0.06).
